# Device and Circuit Co-Optimization of Split-Controlled Flip-Flops Against Aging Towards Low-Voltage Applications

**DOI:** 10.3390/mi17010111

**Published:** 2026-01-14

**Authors:** Yuexin Zhao, Jingjing Tan, Lin Chen, Hao Zhu, Qingqing Sun

**Affiliations:** 1 College of Integrated Circuits and Micro-Nano Electronics, Fudan University, Shanghai 200433, China; 2 National Integrated Circuit Innovation Center, Shanghai 201203, China

**Keywords:** SCFF, transistor aging, BTI, HCI, transistor-level design

## Abstract

The continued downscaling of transistors has exacerbated aging mechanisms such as bias temperature instability (BTI) and hot-carrier injection (HCI), posing significant reliability challenges for nanoscale integrated circuits. These effects are particularly critical to flip-flops operating at low supply voltages, which are essential for ultra-low-power applications including the Internet of Things (IoT) and biomedical implants. In this work, we address the aging issue in low-voltage Split-Controlled Flip-Flops (SCFFs) by proposing a novel transistor-level mitigation technique specifically tailored to this architecture within a domestic 14 nm process library. Through a detailed analysis of aging-critical transistors, three targeted enhancement strategies are introduced. Simulation results demonstrate that the improved SCFF achieves more than a 60% reduction in PMOS threshold voltage degradation and a 40% reduction in timing delay, while maintaining robust operation at a supply voltage as low as 0.4 V. These results highlight the effectiveness of the proposed approach in mitigating aging effects and enhancing reliability under low-voltage operation.

## 1. Introduction

With the continuous advancement of semiconductor process technology, transistor feature sizes have progressively scaled down toward physical limits. This miniaturization offers significant advantages, including enhanced switching speed, improved data processing capability, and reduced power consumption. However, as technology nodes shrink, aging effects such as bias temperature instability (BTI) and hot-carrier injection (HCI) have become critical reliability concerns [1]. These mechanisms cause threshold voltage (Vth) shifts, mobility degradation, and timing failures, particularly in ultra-low-voltage circuits where timing margins are inherently limited [2,3].

Driven by stringent power constraints and long-term reliability requirements, low-voltage flip-flops are increasingly adopted in a wide range of applications, including IoT sensor nodes, smart labels, environmental monitoring chips for edge intelligence, and implantable medical devices in biomedical electronics. Among various designs, the split-controlled flip-flop (SCFF) is particularly attractive for ultra-low-power scenarios due to its ultra-low power consumption, wide voltage adaptability, and non-redundant switching characteristics [4]. However, aging effects in flip-flops are significantly aggravated under low-voltage conditions. In low-voltage design, circuit speed decreases and initial timing margins become extremely limited. The additional delay introduced by aging mechanisms can therefore easily cause timing violations during data sampling and latching, leading to functional failures. Meanwhile, the reduced signal swing at low voltages increases susceptibility to noise, and aging-induced performance degradation further amplifies this sensitivity, substantially raising the risk of erroneous operation. Moreover, PMOS transistors in SCFFs in the feedback loop are subject to prolonged stress during normal operation and thus face severe aging risks dominated by negative bias temperature instability (NBTI) [5,6,7,8]. Consequently, reliability enhancement through transistor sizing optimization, structural modification, or process-level improvements becomes essential.

Previous studies have extensively investigated aging phenomena in conventional flip-flop architectures. For example, a comparative analysis framework was proposed to evaluate aging effects across four representative flip-flop structures implemented in both CMOS and FinFET technologies, revealing critical reliability trade-offs [9]. BTI-induced degradation in nanoscale CMOS flip-flops has also been comprehensively assessed to evaluate the impact on key timing parameters such as setup time, hold time, and clock-to-output delay [10]. Furthermore, a dual-threshold voltage design technique has been introduced to mitigate aging-related performance degradation [11].

To date, most existing anti-aging techniques focus on classical flip-flop architectures, which; however, often fail to meet the demands of modern ultra-low-power applications due to intrinsic performance limitations. As a result, recent research on trigger circuits has increasingly emphasized low power consumption and robustness against performance variations, particularly under low-voltage operation [12,13]. The SCFF has been studied as a promising candidate due to its inherent energy efficiency and wide voltage operating range. Nevertheless, achieving ultra-low-voltage operation typically requires applying reverse body bias to lower the effective Vth, introducing additional design complexity and further exposing the circuit to accelerated aging during long-term operation [14]. To date, effective aging mitigation strategies are still not fully explored, especially designs specifically tailored for SCFFs [15,16].

Here, in this work, we focus on enhancing SCFF reliability through transistor-level optimization and structural improvements. In the proposed approach, the SCFF operates over an ultra-wide dynamic voltage range to minimize power consumption. Aging effects induced by HCI are alleviated by shortening the channel length of transistors on critical paths, while key aging-sensitive devices are identified through a detailed structural analysis of BTI- and HCI-induced degradation. By restructuring the flip-flop topology, the effective stress time of critical transistors is significantly reduced, particularly those in the feedback loop. As a result, aging-induced threats such as Vth drift and increased leakage current are effectively mitigated. Under a domestic 14 nm process, simulation and stress test results demonstrate that the degradation rate of key timing parameters is reduced by more than 50%, with modest and controllable power and area overheads. Moreover, the optimized SCFF maintains stable operation under high-frequency stress conditions, meeting the stringent requirements of long-lifetime ultra-low-power applications.

## 2. Aging Effect in SCFF

The SCFF, while designed for low-voltage operation, exhibits pronounced sensitivity to aging. The scaling of transistor dimensions raises serious aging concerns for flip-flop stability and long-term reliability. Among various degradation contributors, BTI and HCI are the most significant aging mechanisms [17]. Their effects are so pronounced that they profoundly influence the system-level performance and power. Effects like BTI and HCI cause performance loss that is severely magnified at low voltages, where timing margins are paramount. Such margins are fundamental to reliability, ensuring signals settle within the clock’s required window. Therefore, a detailed analysis of the SCFF key timing parameters and the dominant aging mechanisms (BTI/HCI) is necessary to pinpoint the critical factors for improvement, which is the focus of the following sections.

### 2.1. Timing Metrics of SCFF

**Figure 1:**
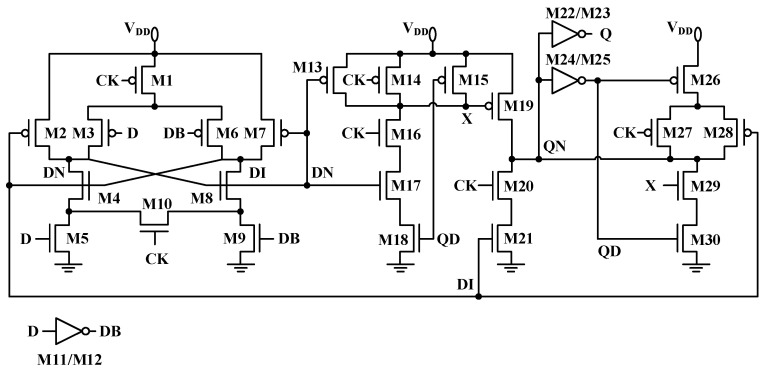
Schematic of the SCFF structure.

This study enhances the aging mitigation capability of the SCFF [4]. The proposed design enables ultra-wide dynamic voltage scaling while reducing both active and leakage power. As shown in Figure 1, transistors M1–M10 form the master latch with a differential structure, while M13–M30 constitute the slave latch, implementing split control functionality. The differential architecture enhances signal stability under ultra-low-voltage conditions, and the slave latch’s split control mechanism optimizes timing response, ensuring reliable SCFF operation in ultra-low-voltage scenarios.

The optimized SCFF maintains a low-power-delay product across process corners and supply voltages. Regarding the timing operation, when the input D is high and the clock is low, the master latch becomes transparent, allowing the signal to propagate to node DN, while the slave latch is cut-off. Conversely, when D remains high and CLK transitions high, the data are transferred from DN to the output Q. A feedback loop subsequently maintains the low value at DN, thereby preventing signal crosstalk or errors.

Flip-flops are widely used in pipelined architectures, and their timing characteristics typically determine the maximum operating frequency [18]. Therefore, to evaluate the effectiveness of the SCFF aging optimization, it is essential to measure key timing parameters, including setup time (t_setup_), hold time (t_hold_), clock-to-output delay (t_cq_), input-to-output delay of the master latch, and the overall input-to-output delay of the SCFF. These parameters significantly impact data transmission efficiency and pipeline stability. When the aging duration exceeds a critical threshold, the accumulated delay impairs the circuit ability to accurately sample data, leading to potential functional errors.

**Figure 2:**
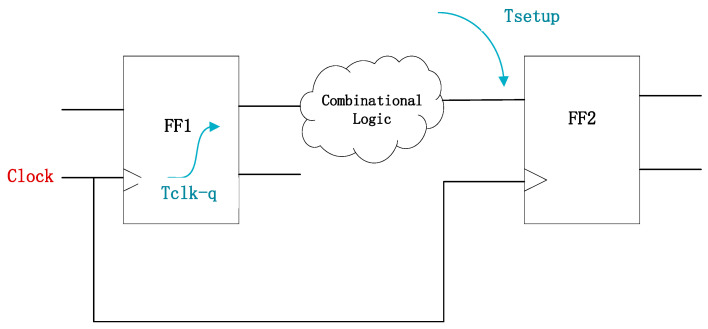
Illustration of the timing constraints for the flip-flop.

Before implementing aging optimization, flip-flop timing metrics must be defined as evaluation criteria. When analyzing flip-flop timing delays, these parameters must be thoroughly considered to ensure long-term reliable operation. To guarantee the correct functionality of digital circuits, flip-flops impose strict timing constraints on input data. The setup time requires data to remain stable before the arrival of the clock edge [19], while the hold time mandates data stability after the clock edge, as shown in Figure 2. These constraints, especially the clock-to-output delay (t_cq_), require rigorous verification during digital design. If aging causes t_setup_ to exceed the allowable clock cycle range, data sampling errors may occur. The correction of timing violations in digital circuit design commonly employs techniques such as buffer insertion or an increase in the local supply voltage. However, these measures introduce additional capacitive loads and switching activity, ultimately increasing dynamic power consumption [20]. Therefore, strict timing adherence is essential to prevent violations and data corruption in pipelined processors and high-speed interfaces.

Failure to effectively mitigate transistor aging can lead to aging-induced circuit failures. Common failure modes in digital circuits, such as metastability and timing violations, are critical factors that severely degrade system timing performance. Transistor aging degrades the timing performance of flip-flops and considerably elevates overall power consumption. This increase in power consumption manifests in two ways: on the one hand, static power rises due to elevated leakage currents; on the other hand, switching power is augmented as a result of threshold voltage drift. The dynamic power consumption in flip-flops arises mainly from the switching activity that charges and discharges parasitic capacitance. This power can be modeled by the formula P = C × V^2^ × f, where C is the load capacitance, V is the supply voltage, and f is the switching frequency.

Specifically, increased Vth reduces drain–source current (I_ds_), prolonging the capacitance charging and discharging time. To ensure the flip-flop completes state transitions within clock constraints, a higher drive voltage or relaxed timing constraints may be required. Such an operation will directly increase the V^2^ term in the power formula, leading to higher dynamic power consumption. For high-speed flip-flops, Vth drift reduces switching efficiency, increasing energy consumption per unit time.

Moreover, NBTI and HCI asymmetrically affect PMOS and NMOS transistors: NBTI primarily degrades PMOS, while HCI predominantly impacts NMOS. This disrupts the balance between rising edge and falling edge switching speeds. During state transitions, the charging and discharging transition times, specifically the rise and fall times of internal nodes such as the outputs of the master and slave latches, are prolonged.

Extended transition times contribute to two key power-related issues. One primary effect of prolonged transition times is an increased duration of short-circuit current flowing between the power supply and ground during switching. Although typically a minor component of dynamic power, the cumulative effect of this current under high-frequency operation significantly increases total power consumption. Moreover, slower signal transitions can lead to timing misalignment with adjacent logic blocks. This may induce redundant signal transitions, such as glitches, thereby further elevating dynamic power consumption.

### 2.2. BTI-Induced Aging Mechanism

Bias Temperature Instability, particularly NBTI in PMOS transistors, poses a critical reliability challenge [21]. This phenomenon significantly contributes to flip-flop aging and fundamentally limits the long-term stability of SCFFs. The emergence of this degradation mechanism can be attributed to two primary technological drivers. On the one hand, it is primarily attributed to the aggressive scaling of MOS device dimensions. On the other hand, it results from sustained operational demands in flip-flop circuits. These factors collectively induce elevated transistor temperatures during operation, which accelerates the dissociation of Si-H bonds at the Si/SiO_2_ interface. Consequently, the release and subsequent drift of hydrogen species toward the gate dielectric generate interface traps and oxide charges, ultimately resulting in a progressive shift in the threshold voltage of MOS devices.

BTI degradation and temperature rise are mutually reinforcing. High temperatures accelerate the breaking of Si-H bonds and the diffusion of hydrogen species, exacerbating the BTI effect. The higher the temperature, the faster the BTI degradation rate. When the transistor is in the on-state, also known as the stress phase, its threshold voltage experiences a continuous increase. However, this shift is reversible, and recovery begins once the gate voltage is removed. NBTI significantly influences transistor aging by causing performance degradation under negative gate bias. Thus, reducing the stress time of PMOS without affecting performance is crucial.

The reaction–diffusion model explains the physical mechanism of BTI through chemical reaction and diffusion processes [22]. Specifically, it accounts for the generation of interface defects and the consequent threshold voltage drift observed in NBTI. Although the interpretation of the reaction–diffusion model is very accurate, its computational complexity is relatively high. In this paper, the QSA formula is used to analyze the influencing factors of threshold voltage shift caused by the BTI effect. The QSA formula simplifies complex physical formulas by introducing empirical parameters and simplifying assumptions, as shown as Equation (1) [23]:

(1) $$\Delta V_{th}={A\cdot (V_{gs}-V_{th0})}^{n}\cdot t^{m}\cdot e^{-\frac{E_{a}}{kT}}$$

where *A* is a process-dependent scale factor reflecting gate dielectric quality, interface properties, and material characteristics; $V_{gs}$ is the gate–source voltage (bias voltage); $V_{th0}$ is the initial threshold voltage; *t* is the stress time; and $e^{-\frac{E_{a}}{kT}}$ describes the temperature acceleration effect. High bias voltage enhances the gate electric field, accelerating interface state defect generation and charge trapping. The acceleration effect of bias voltage on BTI effect is described by these terms. High temperature significantly increases the formation rate of interfacial defects. From this formula, it is evident that under constant technology and temperature conditions, reducing transistor stress time can mitigate BTI-induced threshold voltage increase. Therefore, by modifying the feedback circuit structure in the master–slave latch, the conduction time of transistors that are subjected to prolonged stress can be reduced. By modifying the circuit structure, reducing the value of the stress time *t* in Equation (1) is an effective way to alleviate aging effects.

### 2.3. HCI Impact on Critical Transistors

Unlike BTI, HCI poses a more severe long-term reliability challenge due to its irreversible degradation mechanism. The permanent nature of HCI-induced damage leads to cumulative parametric shifts that exceed design margins, ultimately causing premature circuit failure. This phenomenon is particularly critical in advanced nodes.

The HCI mechanism begins when high-energy carriers, such as electrons or holes, are injected into the gate dielectric. This process generates interface state defects and charge trapping. With shrinking transistor channel lengths and increasing operating frequencies, HCI also affects threshold voltage drift. The impact of HCI-induced threshold voltage drift can be analyzed by Equation (2) [24]:

(2) $${\Delta V}_{th\_HCI}={\left((W_{g}\times \frac{1}{H_{g}}(\frac{I_{gate}}{W})\right)}^{m_{g}}+\left(1-W_{g}\right)\times \frac{I_{ds}}{HW}\times {\left(\frac{I_{sub}}{I_{ds}}\right)}^{m})\times (t{)}^{n_{1}}$$

where $W_{g}$ represents the weighting coefficient of gate current and channel hot-carrier injection in this effect. It means that the degradation in HCI can be caused by gate current when $W_{g}$ = 1 or by channel hot-carrier injection when $W_{g}$ = 0. $H_{g}$ and H represent the normalization of the electric field intensities for the gate region and the drain region, respectively; $I_{gate}$, $I_{ds}$, and $I_{sub}$ describe the gate current, drain current, and base current, respectively. $n_{1}$ represents the time index, indicating that the threshold drift increases exponentially with stress time; $m_{g}$ and *m* represent the index factors, indicating the current acceleration effect indices of the gate current mechanism and the drain region mechanism, respectively; *t* is the stress time, and *W* represents the device width.

As the temperature rises, $I_{gate}$ and $I_{sub}$ will also increase accordingly. In addition, $n_{1}$ will also experience increased defect formation and migration due to the elevated temperature. The final result is that the threshold drift rate will significantly increase, and the influence of the HCI effect on device lifespan becomes more sensitive under high-temperature conditions.

## 3. Anti-Aging Strategies

### 3.1. Critical Transistors Affecting Aging

Before optimization, transistor aging state can be simulated by increasing the gate voltage, primarily for PMOS transistors. Simulations were performed using PMOS transistors with a drawn gate length of 16 nm and a poly finger width of 922 nm, resulting in a transistor width-to-length ratio of 57.625. As shown in Table 1, for flip-flop transistors, voltage values equivalent to ten years of aging are applied to all PMOS transistors to account for threshold voltage shifts. After applying the aging-induced voltage offsets to the gates of all PMOS transistors, each PMOS transistor in the master–slave circuit is analyzed individually by removing the previously applied voltage values. This allows for a precise simulation of the delay effect of a single transistor on the overall performance of the flip-flop.

To identify which transistors are most critical to circuit aging, a method based on comparative delay analysis is employed. This is achieved by comparing the circuit delay after individually shifting each transistor’s Vth with the baseline delay obtained from the simultaneous aging of all transistors. Transistors whose individual aging-induced delay closely approximates the full-circuit aging delay are identified as significant contributors.

Using this method, three key timing parameters were analyzed following the removal of gate voltage shifts from individual PMOS transistors: the clock-to-output delay, the data-to-complementary-data-node delay, and the data-to-output delay. This analysis identified M3 and M6 as having a significant impact on main latch performance degradation.

### 3.2. Reducing the BTI Effect Through Structural Modification

Changes in transistor structure mainly affect the BTI effect, and the impact on PMOS transistors is much greater than that on NMOS transistors. Therefore, before structural improvement, aging was simulated by increasing the PMOS gate threshold voltage (ΔV_th_). After simulated aging, key timing parameters (D-DN delay, CLK-Q delay, and D-Q delay) were compared.

Before improving the circuit structure, it is first necessary to identify the PMOS transistor that has the most significant impact on the circuit, as determined in the previous section. Additionally, during the circuit analysis process, a recovery loop with an excessively long stress time was identified. By modifying the circuit structure, stress time along the critical path can be reduced. According to the QSA formula, reducing PMOS conduction time is the most effective strategy for mitigating BTI-induced aging.

The primary goal of structural modification is to minimize PMOS stress time without compromising flip-flop functionality. In master–slave flip-flops, the feedforward path typically serves as the data transmission route from input to master output, and from flip-flop input to slave output. Thus, structural improvements aimed at reducing PMOS stress time along the critical path have limited effectiveness in such configurations.

#### 3.2.1. Optimization of Sensitive PMOS Transistor Conduction Time

In the conventional SCFF main latch structure, PMOS transistors suffer from non-essential conduction. For example, M3 remains on when CLK = 1 and D = 0, leading to prolonged NBTI stress accumulation. Consequently, V_thp_ exhibits a forward drift of 23.2764 mV, contributing 64.5% of the total degradation. Due to persistent conduction, the stress duration of M3 accounts for 50% of the operational time, significantly accelerating aging.

Conditional conduction of M3/M6 is achieved by introducing a bridge transistor network to reconstruct the main latch data path. Adding a clock-controlled bridge transistor between DNR/DN and DIL/DI paths enables logical decoupling, allowing M3 to be on only when CLK = 0 and D = 0, and M6 to be on only when CLK = 0 and D = 1. When CLK = 1, the bridge transistor conducts, maintaining the static pull-down state of DI/DN to prevent data loss. The ΔV_thp_ of M3 decreased from 23.2764 mV to 5.007 mV, with an improvement rate of 78.5%. The conduction time ratio dropped from 50% to 25%, and the D-DN rising edge delay decreased from 21.788 ps to 20.425 ps. Power consumption increased by 8%, while the area increased by only 4%.

Aging was simulated by increasing threshold voltage degradation at each PMOS gate. M3 and M6 were identified as having a significant impact on circuit aging. In the original circuit, M3 and M6 conduct unnecessarily when CLK = 1 (DN = 1, D = 0). Bridge transistors were added between DNR/DN and DIL/DI to ensure that M3/M6 conduct only when CLK = 0 (D = 0 → M3 on, D = 1 → M6 on). Simulation shows that the M3 conduction time decreased from 50% to 25%, and ΔV_th_ degradation decreased from 23.2764 mV to 5.007 mV (a 78% reduction).

#### 3.2.2. Reconstruction of the Data Recovery Loop of the Main Latch

From the SCFF topology, it can be observed that to achieve low power consumption, the holding circuit and forward circuit of the main flip-flop share transistors M4, M5, M8, and M9. If the aforementioned circuit structure is directly modified, there is a potential risk of affecting the circuit functionality to some extent. Therefore, in the proposed modification strategy, the feedback loop containing transistors M2 and M7 was selected for modification.

Transistors M2 and M7 are connected in series in the pull-down network. The gate of M2 is controlled by the DI signal, while M7 is controlled by DN. Consequently, when input data is 0, M2 remains continuously on; similarly, when input data is 1, M7 remains on. For the entire main latch, regardless of the input data value, one PMOS transistor in the recovery loop is always conductive. As shown in Equation (1), when the flip-flop stores data with equal probability, BTI degradation can be mitigated by reducing the conduction probability of M2 and M7 to 0.5.

In the original circuit, delayed signals DI and DN control M2 and M7, resulting in prolonged conduction time. When the clock signal is low, DI and DN are generated by D and DB signals conducting through the pull-up network, introducing additional conduction time due to propagation delay. To minimize unnecessary conduction time, M2 and M7 gate signals can be replaced with direct D and DB inputs, eliminating delay-induced conduction. Additionally, a PMOS transistor controlled by the clock signal must be inserted in series with the pulse clock transistor to ensure turn-off when the clock signal is high.

Separately inserting a PMOS transistor for M2 and M7 would significantly increase power consumption and introduce additional delay. Therefore, the existing PMOS transistor whose gate is controlled by CLK is shared to enable logical control of the CLK signal over the conduction time of M2 and M7.

Thus, the structural improvement for the main flip-flop is as follows: the gates of M2 and M7 are directly controlled by the input signals D and DB, respectively, while sharing the original PMOS transistor controlled by CLK. This configuration allows M2 to be off when CLK = 0 and D = 0, and M7 to be off when CLK = 0 and D = 1. After optimization, redundant switching of PMOS transistors is reduced, leading to lower dynamic power consumption and a decreased aging rate.

#### 3.2.3. Stress Dispersion Design of the Slave Latch

The slave latch (M13–M30) achieves low power consumption through split control, with the critical path being D → DN → QN → Q. M24 to M30 are used as feedback loops for data storage. When analyzing the slave latch recovery circuit structure, it was found that the gates of M26 and M30 in the original circuit were driven by QD. M26 and M30 are two transistors in the feedback loop of the slave latch. When CLK is low, the input signal D passes through the main latch to the slave latch section, and M26 and M30 are in the conducting state as part of the feedback loop. When CLK is high, the slave latch transmits the data from the main latch to the output signal Q. During this time, the feedback loop is not conducting. However, transistors M26 and M30 are driven by the signal QD and were originally stressed for 50% of the cycle, equivalent to one half-cycle of the clock. From the QSA formula, the α coefficient is 0.5. This increases the time that the transistors are in the stress state and exacerbates the aging effects. Similarly, to reduce conduction time, operations similar to Improvement 2 are needed. The circuit structure needs to be modified so that when CLK = 1, the time that the feedback loop is in the stress state is reduced. The gates of M26/M30 can be connected in series with a pulse clock-controlled transistor to achieve turn-off when CLK = 1. The improved feedback circuit of the slave latch comprises transistors M22 to M27. When CLK = 1, transistor M23 is in the cut-off state. When QD = 1, since M24 and M26 are not conducting, M22 is also in the cut-off state; the same applies when QD = 0. When CLK = 0, the feedback loop conducts normally, and the output Q remains stable.

**Figure 3:**
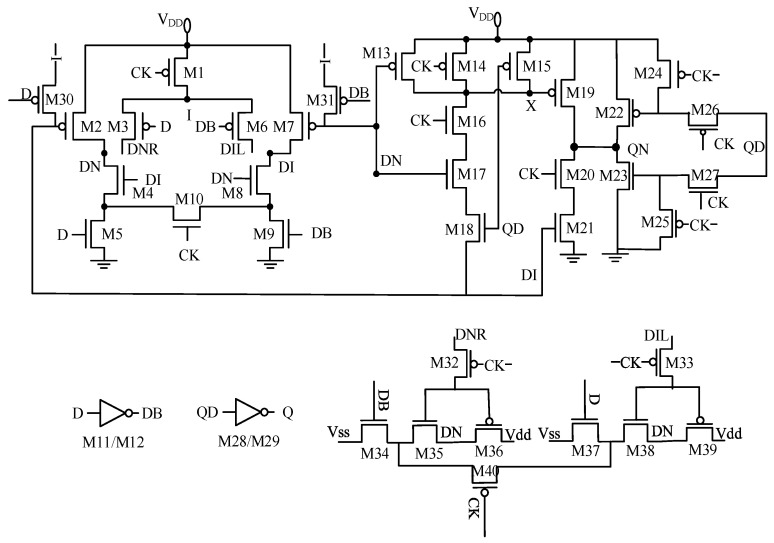
Circuit diagram after structural optimization.

The recovery circuit was modified to ensure that M26 and M30 conduct only when CLK is low and QD changes, thereby reducing their stress time to 25% of the cycle. Furthermore, an NMOS pull-down path was added to guarantee a static shutdown during the high phase of the clock. As a result, the threshold voltage degradation of M27 decreased from 22.379 mV to 17.124 mV, and the contribution of hot-carrier injection to this degradation dropped from 62.8% to 10.2%. The circuit diagrams after optimization are shown in Figure 3.

### 3.3. Transistor-Level Structure Reconfiguration

Notable differences exist in aging mechanisms between PMOS and NMOS transistors. For PMOS devices, the dominant aging mechanism is NBTI-induced positive threshold voltage shift, occurring under negative gate bias and high temperatures and following a time-dependent power-law model. For NMOS devices, HCI is the primary aging mechanism, becoming significant under high-frequency switching or high drain voltage conditions, with degradation proportional to channel current density.

As discussed previously, circuit-level structural optimizations mitigate BTI-induced aging. In SCFFs, while BTI is a major aging factor, HCI also has a considerable influence. Specifically, HCI in NMOS transistors (e.g., M5 in the clock path) under high drain voltage conditions causes forward Vth shift, increasing the Clk-q delay.

**Figure 4:**
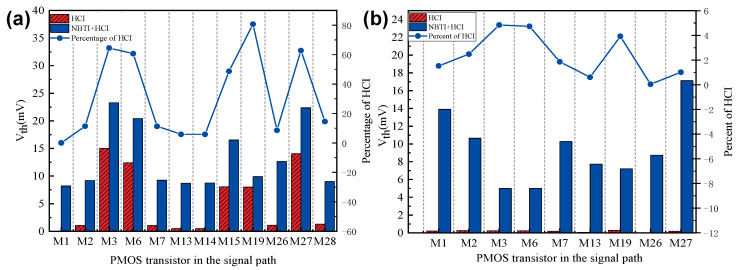
Comparison of circuit performance degradation due to HCI and BTI effects in PMOS transistors (**a**) before and (**b**) after optimization.

However, the degradation in PMOS transistors is an order of magnitude higher than that in NMOS transistors in the SCFF circuit. Therefore, our research focuses on HCI-sensitive PMOS transistors rather than NMOS transistors. In the simulation, the aging delay of PMOS transistors with the mentioned structure improvement was analyzed, and the proportion of the HCI effect in the overall Vth degradation was calculated. By analyzing the ratio of HCI-induced threshold voltage shift to total variation, as shown in Figure 4a, it is observed that the HCI trap density in M19 and M27 (in the latch feedback path) increases significantly under high-frequency clock stress. HCI-induced degradation exceeds 80%, indicating pronounced aging effects and necessitating targeted optimization.

Therefore, process optimizations for HCI-sensitive PMOS transistors are essential. Based on the analysis of the QSA formula, increasing the gate oxide thickness for PMOS transistors in high-frequency critical paths is an effective strategy to reduce HCI sensitivity. A larger transistor width effectively reduces the current density and peak electric field near the drain, thereby mitigating hot-carrier degradation. Consequently, the threshold voltage shift due to HCI exhibits an inverse dependence on device width. Within the SMIC 14 nm process library, increasing the transistor width-to-length ratio can effectively reduce the influence of the HCI effect on threshold voltage offset. The HCI effect of the improved PMOS transistor is significantly enhanced, as shown in Figure 4b.

## 4. Results and Discussion

To evaluate the effectiveness of the proposed SCFF modification, a comprehensive simulation study was conducted under varying supply voltages and accelerated aging conditions. The results demonstrate that the improved circuit maintains full functional integrity while achieving a significant enhancement in aging resilience, a conclusion further verified through HSPICE simulations and process corner analysis performed under domestic 14 nm process conditions.

**Figure 5:**
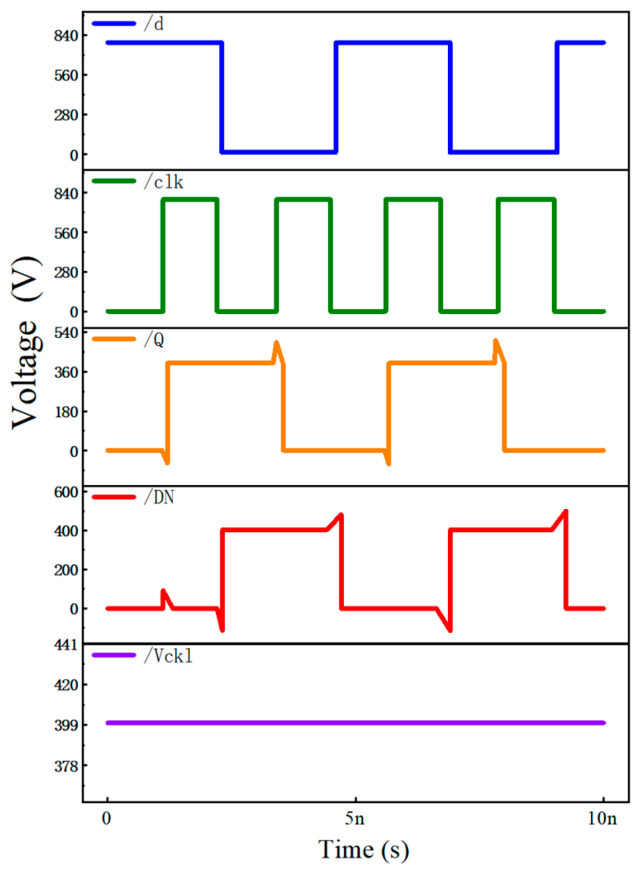
Waveform diagram showing voltage versus time.

The improved SCFF operates correctly at Vdd = 0.4 V (as shown in the enhanced waveform diagram), with the rising edge delay of DN/DI reduced from 3.378 ns to 0.365 ns, validating the enhancement in timing reliability, as shown in Figure 5.

The proposed structural modifications targeted the reduction in stress duration in critical PMOS transistors (M3 and M6). By decoupling their conduction phases and optimizing the data recovery loop, unnecessary stress time was halved. This resulted in a 31% reduction in aging-induced delay for the 0→1 transition and improved symmetry in delay degradation between the 0→1 and 1→0 transitions. The modified SCFF maintained stable operation at 400 mV, validating suitability for ultra-low-voltage applications. The QSA model was adopted for SCFF aging simulation in this study. The aging conditions were set to a temperature of 125 °C and a duration equivalent to ten years. Performance differences between original and improved structures were compared via HSPICE simulation.

First, significant inhibition of threshold voltage degradation is observed. The NBTI degradation of the key PMOS transistors, namely M3 and M6, was reduced from 23.2764 mV to 5.007 mV, which represents an improvement of 78.5%. Meanwhile, the HCI degradation in the NMOS transistor M27 decreased from 22.379 mV to 17.1243 mV, corresponding to a 23.4% improvement. In addition, comprehensive improvement in timing performance is achieved. The proposed design yielded substantial timing improvements while operating at 0.4 V. The setup time showed a reduction of 61.6%, from 89.63 ps to 34.40 ps, and the clock-to-output delay was decreased by 74.6%, with no compromise in logical functionality.

By comparing the performance of the original SCFF, the gate length-adjusted SCFF (SCFF-GL), and the structurally modified SCFF (SCFF-Mod), it was found that SCFF-Mod achieves the lowest aging delay increment (0.84 ps for 0 → 1 transition) at the cost of a 20% increase in PDP. In contrast, SCFF-GL showed limited improvement due to increased RC delays in the feedforward path.

Aging analysis also identified key transistors contributing most to HCI degradation. Through the QSA model, HCI impact can be mitigated by increasing transistor gate length. Under typical conditions (Vdd = 0.4 V), the degradation increment decreased by approximately 30%. The improved SCFF reduced the aging delay increment by 55% in the worst case, with PDP increased by only 20%.

The SCFF-Mod was evaluated across process corners (tt, ss, ff, fnsp, snfp). The aging delay increment was consistently reduced, with the most notable improvement under the ss corner (55.5% reduction). Structural robustness to process variations underscores its practicality for wide range voltage scaling. At Vdd = 400 mV, the SCFF-Mod demonstrated correct functionality across all test vectors, confirming reliability in sub-threshold operation. The split-controlled design eliminated redundant switching, contributing to PDP efficiency.

Table 2 compares the anti-aging performance of the modified flip-flop with several classic low-voltage flip-flop structures and analyze the optimization effect in terms of performance, power consumption, and area [10,25]. The d2q delay is the average value of the 0-to-1 and 1-to-0 output propagation delays. The propagation delay of the SCFF declines by approximately around 46.02%, showing a significant decline in transmission delay. Among all the evaluated FFs, the SCFF shows the most pronounced decrease in propagation delay, highlighting its superior performance. However, the number of transistors in our work has increased significantly by 33%, and the power consumption has also increased by 26.31% due to the extra transistors when restricting the circuit. Overall, the performance of the improved SCFF is significantly enhanced, accompanied by increases in area and power consumption. The optimized structure has a very obvious improvement in the delay of the trigger with controllable power and area overheads.

The proposed modifications successfully address aging effects while preserving ultra-low-voltage capabilities. By combining transistor-level tuning and structural optimizations, the design achieves a balanced trade-off between aging resilience and power efficiency. Results validate the SCFF’s suitability for energy-critical applications requiring long-term reliability.

## 5. Conclusions

In conclusion, this work investigates transistor-level anti-aging strategies for SCFFs under an FinFET process and proposes three effective approaches—conduction time optimization, main latch loop reconstruction, and secondary latch stress dispersion. Using setup time and clock-to-q delay as metrics, the proposed scheme significantly suppresses aging-induced delay, achieving reductions of 61.6% for the 0 → 1 transition and 74.6% for the 1 → 0 transition at the FF corner, while maintaining consistent effectiveness across all process corners, demonstrating strong robustness to process variation and aging. Experimental results confirm that the optimized SCFF effectively mitigates threshold voltage drift and leakage increase, reduces PMOS degradation by over 60%, improves timing delay by more than 40%, and supports stable operation at 0.4 V under high-frequency stress with controllable power and area overheads. Although the introduction of redundant transistors and bias circuits increases layout area by about 30% and adds some dynamic power consumption, the scheme still outperforms conventional redundancy methods, offering a practical solution for long-life, low-power nanoscale IC design. Future work will point toward device-level parallel redundancy for further enhancing aging resilience.

The presented NBTI analysis demonstrates that integrating reliability considerations into circuit design can effectively mitigate aging effects. Although such techniques may introduce power and design overheads, they provide a vital foundation for future robust and long-lifespan circuit architectures. In future analyses and optimization of subsequent flip-flop structures, particular attention should be paid to the aging impact of NBTI on circuit behavior to further enhance semiconductor device performance while providing comprehensive reliability evaluation.

## Figures and Tables

**Table 1:** Measured aging delay distribution across PMOS devices in the SCFF.

	Clk-to-Q Delay (ps)		Setup Time (ps)		Data-to-Q Delay (ps)	
	0 → 1	1 → 0	0 → 1	1 → 0	0 → 1	1 → 0
Original Circuit	14.112	21.67	90.122	49.469	104.234	71.139
Aged Circuit	14.535	21.788	92.984	52.713	107.519	74.501
M1	14.534	21.788	92.95	52.687	107.484	74.475
M2	14.535	21.79	93.475	52.71	108.01	74.5
M3	14.535	21.788	92.982	49.427	107.517	71.215
M6	14.53	21.788	89.606	52.715	104.136	74.503
M7	14.53	21.788	93.04	52.755	107.57	74.543
M13	14.53	21.789	93.012	52.751	107.542	74.54
M14	14.535	21.841	92.984	52.713	107.519	74.554
M15	14.515	21.79	92.984	52.713	107.499	74.503
M19	14.536	21.619	92.984	52.713	107.52	74.332
M26	14.534	21.788	92.984	52.71	107.518	74.498
M27	14.533	21.788	92.936	52.698	107.469	74.486
M28	14.534	21.798	93.056	52.714	107.59	74.512

**Table 2:** Comparison of performance among various structures.

Structure	States	td2q(ps)	(Δtd2q) × 100	Transistor Count	ΔNumber	Average Power (nw)	(ΔPower) × 100
TGFF	Original	149.52	−5.73	22	4	322.03	7.44
	Modified	140.95		26		346.01	
TGFFV2	Original	155.98	−10.37	22	4	347.35	−0.38
	Modified	139.8		26		346.01	
C2MOS	Original	146.55	−2.66	20	4	591.14	−2.11
	Modified	142.65		24		578.62	
HLFF	Original	63.12	−12.21	20	7	450	35.55
	Modified	55.41		27		610	
out work-SCFF	Original	91.01	−46.02	30	10	503.2	26.31
	Modified	44.99		40		635.6	

## Data Availability

The data presented in this study are available on request from the corresponding author.
